# Healthcare payment rules and household asset allocation: evidence from China's DRG/DIP reform

**DOI:** 10.3389/fpubh.2026.1949561

**Published:** 2026-08-26

**Authors:** Jiyang Li, Rui Pan

**Affiliations:** 1 School of Economics and Management, Wuhan University, Wuhan, China; 2 School of Social Sciences, Tsinghua University, Beijing, China

**Keywords:** DRG/DIP payment reform, household financial asset allocation, medical expenditure uncertainty, precautionary saving, staggered difference-in-differences

## Abstract

**Objective:**

This study examines whether China's diagnosis-related group/diagnosis-intervention packet (DRG/DIP) healthcare payment reform changes household financial asset allocation and explores the institutional and household pathways associated with that response.

**Methods:**

We link official 2019 and 2021 pilot designations to five waves of the China Family Panel Studies (CFPS) from 2014 to 2022 and estimate a staggered difference-in-differences model with individual and year fixed effects and city-clustered standard errors.

**Results:**

In the fully adjusted specification, reform is associated with a 0.3952 increase in ln(1+household financial product value) (SE = 0.1301; *p* < 0.01), equivalent to an approximately 48.5% increase in 1+reported portfolio value. The result is robust to PSM-DID, an inverse hyperbolic sine transformation, exclusion of cross-city movers, and 5,000 city-level placebo permutations. In a pre-trend diagnostic comparing the 2021 cohort with never-treated cities, the 2014, 2016, and 2018 coefficients are jointly indistinguishable from zero [F(3, 64) = 0.442; *p* = 0.724]. Exploratory two-step tests are consistent with changes in household consumption expenditure, the mode of government intervention, and industrial upgrading; the estimated effect is stronger among households without a spouse, lower-expenditure households, and households with internet access.

**Conclusion:**

The findings suggest that healthcare payment rules may have household-welfare spillovers by improving the capacity to cope with medical expenditure risk and strengthening household financial resilience. The mechanism results are interpreted as channel-consistent evidence rather than causal mediation.

## 1 Introduction

As household incomes rise and financial markets develop, household financial asset allocation increasingly affects property income, consumption capacity, and economic welfare. Compared with housing and other real assets, however, Chinese households continue to exhibit relatively low participation in financial markets, concentrated portfolios, and limited holdings of risky assets. In addition to conventional determinants such as income, education, age, and household structure, medical expenditure risk and its institutional setting are important influences on household financial decisions. Health shocks are uncertain in timing, difficult to predict in scale, and often create immediate liquidity needs. When future medical expenditure cannot be assessed accurately, households tend to hold larger liquid reserves and adopt more conservative portfolios. Medical insurance institutions therefore affect not only access to care and the efficiency of insurance funds, but also consumption, saving, and financial product holdings through expectations of future expenditure risk.

For many years, China's basic medical insurance system primarily reimbursed providers on a fee-for-service basis, closely linking provider revenue to the number of services delivered. Under this arrangement, medical institutions could increase revenue by expanding tests, treatments, and consumable use, while insurance authorities faced informational constraints in identifying, monitoring, and controlling medical costs. The shift from fee-for-service to payment by disease group or disease category redesigns both provider incentives and the governance rules of medical insurance funds. By setting payment standards at the group or category level, DRG/DIP reform redirects provider incentives from service volume toward treatment cost, service quality, and case mix, thereby increasing providers' responsibility for cost control. The reform may consequently improve service efficiency and make medical cost formation more transparent and predictable, altering household expectations of future medical burdens and institutional stability.

DRG and DIP are prospective provider-payment arrangements embedded in China's basic medical insurance system. They regulate settlement between medical insurance agencies and healthcare providers rather than changing households' eligibility for basic coverage. DRG classifies inpatient cases by diagnosis, treatment, patient characteristics, and expected resource use, whereas DIP forms disease-treatment combinations and assigns points within a regional global budget. Both approaches weaken the direct link between provider revenue and service volume and strengthen providers' responsibility for cost and quality. China's reform progressed from the 2017 pilots in Sanming, Shenzhen, and Karamay to the 2019 national DRG pilot cohort and the subsequent DIP program. In 2021, pilot regions entered actual payment and the Three-Year Action Plan established the objective of broad coverage across coordinating regions, medical institutions, disease categories, and medical insurance funds. This sequence moved payment reform from local experimentation to an institutional component of basic medical insurance governance.

As implementation expanded, research increasingly examined the internal effects of DRG/DIP reform on the healthcare system. Jiang et al. evaluated medical service efficiency from the perspective of resource inputs and outputs and documented substantial regional differences in efficiency and its determinants (1). Chen et al. compared the implementation and effects of DRG and DIP through bibliometric analysis and showed that the literature mainly addresses medical cost control, shorter hospital stays, service efficiency, and quality of care (2). Using city-level medical institution data, Jiang et al. found that average cost per visit, length of stay, and case mix are closely related to DIP settlement payment rates (3). Xu et al. documented differences in medical costs and case mix across treatment locations and demonstrated the practical need to incorporate cross-regional care within a province into a unified payment system (4). Drawing on international experience, Cao et al. concluded that prospective payment can standardize treatment and strengthen cost control, while also creating risks such as avoidance of severe cases, patient selection, and coding adjustment (5). Although this literature provides substantial evidence on providers, medical costs, and insurance funds, it pays limited attention to the downstream effects of payment reform on household economic decisions.

A related literature investigates how health risk and medical protection affect household behavior. Yang showed that different forms of health risk may affect household consumption through precautionary saving and illness shocks (6). Ding and Feng further demonstrated, using urban household data, that health risk is an important determinant of both the level and composition of consumption (7). Li et al. found that uncertainty about medical expenditure suppresses consumption among older households, whereas compensation through social medical insurance partly mitigates this effect (8). At the regional level, Guo documented marked temporal and spatial variation in precautionary saving motives among Chinese residents (9). These studies establish that medical expenditure risk and the degree of insurance protection influence intertemporal resource allocation, but they focus mainly on consumption and saving rather than on whether institutional change induces households to reallocate wealth toward financial products.

Research on household finance explains portfolio differences primarily through social protection, demographic structure, and risk perception. Tian and Huang found that basic pension insurance affects both housing wealth and household financial asset allocation (10). Cai argued that population aging, risk-bearing capacity, and retirement needs jointly shape household financial choices (11). Zheng emphasized that households balance safety, return, and liquidity when selecting financial products (12). This work identifies multiple determinants of household portfolios, yet the potential spillover of healthcare payment rules, as an institution of public governance, into household financial behavior remains insufficiently examined.

Against this background, we treat DRG/DIP reform as a city-level quasi-natural experiment and match the reform schedule to CFPS panel data. A staggered difference-in-differences model is used to estimate the effect of reform on the total value of household financial products. The analysis addresses three questions. First, does DRG/DIP reform significantly change household financial product holdings in pilot cities? Second, if an effect exists, is it associated with household consumption expectations, the mode of government intervention, and changes in the urban industrial structure? Third, do households with different marital status, expenditure levels, and digital access respond equally to the institutional change?

This study makes three contributions relative to the three strands of literature reviewed above. First, compared with studies centered on providers, medical costs, and insurance-fund performance, it extends the outcome domain to household financial asset allocation and identifies a potential cross-domain spillover of payment rules. Second, compared with research on health risk, precautionary saving, and consumption, it examines whether a city-level institutional reform changes a distinct household balance-sheet outcome. Third, compared with household-finance studies focused on social protection and demographics, it links city-level policy exposure to individual longitudinal CFPS data and examines theoretically motivated differences by household responsibility, budget constraints, and digital access. Each empirical hypothesis corresponds to a separate subsection of the theoretical framework.

From a public-health perspective, household financial resilience affects whether families can absorb health shocks without sharply reducing essential consumption, liquidating productive assets, or delaying necessary care. The portfolio outcome is therefore not treated solely as an investment measure; it also reflects households' capacity to plan for medical risk within the broader medical security system. By connecting provider-payment rules with medical expenditure expectations, precautionary saving, and household welfare, the study links healthcare financing reform to medical security, health equity, and financial resilience.

## 2 Theoretical analysis and hypotheses

### 2.1 DRG/DIP reform and household financial product allocation

Although DRG and DIP use different case-classification methods, both seek to weaken the direct link between provider revenue and service volume under fee-for-service payment. DRG classifies inpatient cases according to diagnosis, treatment, patient characteristics, and resource use and settles payment according to group-specific standards. DIP uses the full regional case population to form disease combinations based on diagnosis and treatment, with payment determined by disease scores and a regional global budget. Relative to fee-for-service, both systems standardize medical insurance payment rules and require providers to organize care within more explicit payment constraints.

Household financial allocation depends on current disposable resources as well as uncertainty about future income and expenditure. When medical costs are difficult to predict, households tend to retain more low-yield, highly liquid funds to cover sudden illness and large medical bills. By unifying case classification, standardizing treatment pathways, and strengthening cost constraints, DRG/DIP reform may reduce concern about uncontrolled medical cost growth and improve the predictability of future expenditure. Holding income constant, lower expected medical risk reduces the need to maintain unusually large cash reserves and may encourage households to allocate funds to mutual funds, equities, bonds, wealth-management products, and other formal financial instruments.

Payment reform also sends an institutional signal. Moving from ex post itemized settlement to standardized group- or disease-based payment indicates that medical insurance fund management and healthcare governance are becoming more rule-based. A stable and transparent institutional environment can extend household planning horizons and shift decisions from short-term risk defense toward an integrated allocation across consumption, saving, and investment. Zhao and An found that household aging affects consumption and its composition by strengthening precautionary saving motives (13), while Ye and Guan showed that the type and generosity of social protection alter household consumption decisions (14). Accordingly, when DRG/DIP reform improves expectations of medical expenditure and institutional stability, households may reduce excessive defensive saving and increase holdings of formal financial products.

*Hypothesis 1: DRG/DIP payment reform significantly increases the total value of financial products held by households*.

### 2.2 Household consumption expenditure

Consumption reflects both current resource use and household assessments of future income and expenditure risk. When future medical expenditure is highly uncertain, households may reduce discretionary consumption and accumulate precautionary reserves even if current income is unchanged. By standardizing medical services, strengthening expenditure constraints, and improving the efficiency of insurance funds, DRG/DIP reform may alleviate concern about future medical spending and strengthen consumer confidence. Once expectations become more stable, households may increase both current consumption and longer-term financial investment rather than keeping all additional resources in cash.

Yi et al. found that commercial health insurance promotes consumption by weakening precautionary saving motives (15), confirming that mechanisms for sharing medical risk affect intertemporal resource allocation. Wang argued that medical insurance reform influences individual consumption by reducing the medical burden and stabilizing expectations (16). Using CFPS data, Lu et al. showed that comprehensive medical reform upgrades household consumption structure through improved expectations, lower financial vulnerability, and greater trust in government (17). These findings suggest that changes in medical protection may jointly influence consumer confidence and asset allocation.

*Hypothesis 2: DRG/DIP reform improves household consumption expectations and increases household consumption expenditure, thereby creating a potential channel through which financial product allocation rises*.

### 2.3 Mode of government intervention

Medical payment reform is not merely a technical change in settlement; it also changes the mode of public governance. Under fee-for-service, insurance authorities rely heavily on item review, cost inspection, and ex post supervision, leaving substantial scope for administrative intervention and discretion. DRG/DIP reform transforms dispersed item-level review into standardized management under disease groups, disease categories, and global budget rules. It can therefore reduce direct intervention in individual medical acts and redirect governance from process control toward rule-based constraints and outcome evaluation.

This shift can affect household finance through policy certainty. More stable, transparent, and predictable rules enable households to form longer-term expectations and increase their acceptance of formal financial markets and long-horizon products. Hou and Guan found that economic policy uncertainty reduces both the breadth and depth of household financial investment (18), while Si and Duan showed that policy uncertainty affects macroeconomic activity through investment, consumption, and expectations (19). A decline in the government intervention indicator following DRG/DIP reform should therefore not be interpreted simply as government withdrawal. Rather, it reflects a transition from direct administrative control to standardized, rule-based governance.

*Hypothesis 3: DRG/DIP reform promotes a transition toward rule-based government governance and thereby increases household financial product allocation by strengthening institutional certainty*.

### 2.4 Industrial structure upgrading

By changing provider incentives and the allocation of medical resources, DRG/DIP reform may also affect the urban industrial structure. Standardized payment and treatment rules encourage providers to strengthen cost management, information systems, and specialized services, supporting the development of healthcare informatization, health management, commercial insurance, and related producer services. A more standardized medical service market can also direct capital, technology, and labor toward more efficient health and modern service sectors, thereby advancing the urban industrial structure.

Industrial upgrading can affect household financial allocation through employment and financial supply. Expansion of modern services can create specialized employment and additional sources of property income, improving the economic basis for financial market participation. Agglomeration in finance, information services, and health services may also expand the supply of financial products and reduce the cost of obtaining financial information and services. More stable income expectations and easier access to financial services should increase both participation in and the scale of household financial product holdings.

*Hypothesis 4: DRG/DIP reform promotes urban industrial structure upgrading, thereby creating a potential channel for higher household financial product allocation*.

## 3 Materials and Methods

### 3.1 Model specification

Because cities entered DRG/DIP reform at different times, we use a staggered difference-in-differences model to estimate the effect of medical payment reform on household financial product allocation. The model is specified as follows:


$$\begin{array}{l} {\text{Financia}\text{l}}_{\text{ict}}{=\;\alpha \;+\;\beta \;\text{DID}}_{\text{ct}}{+\;\gamma \;\text{X}}_{\text{i}\text{t}}{+\;\delta \;\text{Z}}_{\text{c}\text{t}}{+\;\mu }_{\text{i}}{+\;\lambda }_{\text{t}}{+\;\varepsilon }_{\text{ict}} \end{array}$$ (1)

where i, c, and t denote the individual, city, and year, respectively. Financial_ict is the total value of financial products held by the household of individual i in year t; DID_ct is the DRG/DIP reform indicator; X_it is a vector of individual- and household-level controls; Z_ct is a vector of city-level controls; mu_i denotes individual fixed effects that absorb time-invariant individual characteristics; lambda_t denotes year fixed effects that capture macroeconomic changes and common time trends; and epsilon_ict is the error term. Because the reform is implemented at the city level, standard errors are clustered by city. To show how model specification affects the estimates, we first regress household financial product value on DID alone, without controls or fixed effects. We then add the full set of individual and city controls together with individual and year fixed effects; this is the main specification.

### 3.2 Variable definitions

#### 3.2.1 Dependent variable

The dependent variable is the total current value of financial products reported by the household in CFPS item FT201. The item aggregates all financial products selected in the preceding holding question, including stocks, funds, bonds, financial derivatives, and other reported investment products. It is distinct from cash and deposits, which are recorded separately in FT1, and money owed to the household, which is recorded in FT901. Because FT201 contains many zeros and is strongly right-skewed, we use the natural logarithm of one plus total financial product value:


$$\begin{array}{l} \text{ln\_ft}201\;=\text{ ln}(1\;+\;\text{ft}201) \end{array}$$ (2)

Here, ft201 is the total value of household financial products. A larger ln_ft201 indicates a larger financial product portfolio. Adding one retains households with zero holdings and reduces the influence of extreme values. As a robustness check, we transform the original value using the inverse hyperbolic sine function, which also retains zeros and avoids dependence on the specific treatment of zero values under the logarithmic transformation.

#### 3.2.2 Core explanatory variable

The core explanatory variable is the DRG/DIP payment reform indicator, DID, defined as:


$$\begin{array}{l} {\text{DID}}_{\text{ct}}{=\;\text{Treat}}_{\text{c}}{\text{x Post}}_{\text{c}\text{t}} \end{array}$$ (3)

Pilot status is identified from the official national DRG/DIP notices and implementation lists issued by the National Healthcare Security Administration and related authorities. Treat_c equals one when the respondent's prefecture-level city belongs to the 2019 or 2021 pilot cohort and zero for cities never treated during the sample period. Post_ct equals one in the first CFPS wave observed after the relevant policy year and in subsequent waves (2020 for the 2019 cohort and 2022 for the 2021 cohort). City exposure is assigned from the respondent's current-wave prefecture code after district- and county-level identifiers are harmonized to the prefecture level. Respondents who move are therefore coded according to their current city in each wave. Only five of the 641 individuals in the fully controlled baseline sample change prefectures; excluding them yields a DID coefficient of 0.4068 (SE = 0.1266; *p* = 0.0019), close to the baseline estimate.

#### 3.2.3 Control variables

To reduce omitted-variable bias, we include controls at both the individual/household and city levels.

Individual- and household-level controls are age squared divided by 100, years of education, presence of a spouse, agricultural hukou status, household size, number of older household members, current employment, and the logarithm of total income over the previous 12 months. These variables capture life-cycle position, education, marital and registration status, household burden, labor participation, and income.

City-level controls are the logarithm of GDP per capita, the urbanization rate of the resident population, GDP growth, financial development, the share of the tertiary sector, and the level of informatization. These variables account for differences in economic development, population structure, growth, financial service supply, industrial structure, and digital infrastructure.

### 3.3 Mechanism and heterogeneity variables

Potential mechanisms are examined through household consumption expenditure, the degree of government intervention, and industrial structure upgrading. Consumption expenditure is transformed as ln(1+x) and standardized to measure consumption capacity and expectations. The city-level government intervention indicator is logged and standardized to capture changes in the mode of local government intervention. The industrial structure upgrading index is likewise logged and standardized to reflect movement toward a more advanced urban industrial structure.

Heterogeneity is assessed according to pre-policy spouse status, total household expenditure, and internet use. Total expenditure is divided into high- and low-expenditure groups at the sample median of pre-policy ln(1+expenditure). Interactions between DID and each grouping variable are estimated to test between-group differences directly rather than inferring differences from the significance of separate subgroup regressions.

### 3.4 Data sources and sample construction

The analysis uses the China Family Panel Studies and matches individual and household information to prefecture-level DRG/DIP pilot lists and regional economic indicators. We harmonize variable definitions across the 2014, 2016, 2018, 2020, and 2022 waves, convert district- and county-level location information to prefecture codes, merge the policy and city-control files by prefecture and year, and remove observations with missing outcome, treatment, or control variables. The full merged sample contains 3,134 individual-year observations, 2,007 respondents, and 119 cities, including 16 cities in the 2019 cohort and 15 cities in the 2021 cohort. For the individual fixed-effects specification, we additionally require each respondent to be observed in at least two waves. The resulting common baseline sample contains 1,689 observations, 641 respondents, and 79 cities.

We evaluate robustness in three ways. First, city-level propensity score matching is combined with DID to reduce selection bias in pilot designation. Second, the dependent variable is replaced by the inverse hyperbolic sine transformation of total financial product value. Third, we conduct 5,000 city-level placebo permutations while preserving the number of treated cities in the 2019 and 2021 cohorts to determine whether the baseline effect could arise from random policy assignment.

## 4 Results

### 4.1 Descriptive statistics

Table 1 reports descriptive statistics for the variables in the effective baseline estimation sample. To maintain comparability with the core fixed-effects regression, the descriptive sample retains only individuals observed in at least two waves and with complete controls, yielding 1,689 individual-year observations.

**Table 1:** Descriptive statistics.

Variable	Code	*N*	Mean	SD	Min	Max
Financial product value (log)	ln_ft201	1,689	11.3066	1.6122	0.0000	16.1181
DRG/DIP reform	Did	1,689	0.1954	0.3966	0.0000	1.0000
Age squared/100	age2_100	1,689	26.1560	13.1194	4.0000	88.3600
Years of education	educ	1,689	12.8289	3.3404	0.0000	22.0000
Has spouse	mar	1,689	0.8680	0.3386	0.0000	1.0000
Agricultural hukou	Rural	1,689	0.2925	0.4550	0.0000	1.0000
Household size	fml	1,689	3.2167	1.3523	1.0000	13.0000
Older household members	old_clean	1,689	0.4221	0.7204	0.0000	4.0000
Currently employed	job	1,689	0.6898	0.4627	0.0000	1.0000
Total income (log)	ln_finc	1,689	11.7213	0.9882	0.0000	14.5087
City GDP per capita (log)	c_ln_pgdp	1,689	11.3994	0.5260	9.6711	12.1981
Urbanization rate	c_urban	1,689	0.7845	0.1360	0.4312	0.9283
GDP growth	c_growth	1,689	0.0505	0.0365	−0.2063	0.1112
Financial development	c_finance	1,689	4.2025	1.6790	1.1486	7.6203
Tertiary industry share	c_industry	1,689	0.5724	0.1305	0.2470	0.8387
Informatization	c_information	1,689	0.0278	0.0164	0.0054	0.1751

Statistics are based on the effective sample for the fully controlled baseline regression. Total financial product value and total income are transformed as ln(1+x).

The mean of the logarithm of total household financial product value is 11.3066, with a standard deviation of 1.6122 and a range from 0 to 16.1181, indicating substantial variation in portfolio size. The mean of DID is 0.1954, implying that approximately 19.54% of observations are in treated cities after reform. In the sample, 86.80% of respondents have a spouse, 29.25% hold agricultural hukou, and 68.98% are currently employed. The city-level variables also display meaningful variation, enabling identification after controlling for differences in economic development, urbanization, financial development, industrial structure, and informatization.

### 4.2 Baseline estimates

Table 2 presents the baseline estimates. Column (1) includes only DID and excludes individual and city characteristics as well as fixed effects. Column (2) adds the full set of individual- and city-level controls and includes both individual and year fixed effects. Both columns use city-clustered robust standard errors because the reform is implemented at the city level.

**Table 2:** Baseline estimates.

Variables	(1) No controls or fixed effects	(2) Full controls and fixed effects
DRG/DIP reform (DID)	0.6294^***^	0.3952^***^
	(0.1362)	(0.1301)
age2_100	–	−0.1363
	–	(0.0977)
Educ	–	−0.0724
	–	(0.0575)
Mar	–	−0.5486^***^
	–	(0.1910)
Rural	–	−0.0026
	–	(0.1740)
Fml	–	−0.0084
	–	(0.0563)
Old_clean	–	0.0663
	–	(0.0880)
Job	–	−0.1970
	–	(0.1702)
ln_finc	–	0.0393
	–	(0.0477)
c_ln_pgdp	–	0.2702
	–	(0.6007)
c_urban	–	−1.0235
	–	(2.9389)
c_growth	–	0.2788
	–	(1.1875)
c_finance	–	0.1223
	–	(0.1901)
c_industry	–	−0.4194
	–	(1.9317)
c_information	–	1.9513
	–	(2.1949)
Constant	10.8105^***^	–
	(0.1868)	–
Individual controls	NO	YES
City controls	NO	YES
Individual fixed effects	NO	YES
Year fixed effects	NO	YES
City-clustered standard errors	YES	YES
Observations	3,134	1,689
Cities	119	79
Individuals	–	641
R-squared	0.0189	0.0251

City-clustered robust standard errors are in parentheses. ^***^ denotes statistical significance at the 1% level. The R-squared in column (2) is the within R-squared. Columns (1) and (2) use different samples because of missing controls and fixed-effects estimation requirements.

Table 2 presents the baseline estimates. Column (1), which excludes controls and fixed effects, yields a DID coefficient of 0.6294 (*p* < 0.01). Column (2) adds the full set of individual- and city-level controls together with individual and year fixed effects; the coefficient declines to 0.3952 but remains significant at the 1% level. Because the dependent variable is ln(1+financial product value), exp (0.3952)−1 = 0.4846. Conditional on the included controls and fixed effects, the estimate therefore corresponds to an approximately 48.5% increase in 1+reported portfolio value. It should not be interpreted as a 48.5% increase in yuan holdings for every household, particularly for observations at or near zero. The result supports Hypothesis 1.

#### 4.2.1 Parallel-trends assessment

To assess pre-reform comparability, we conduct a cohort-specific event-study diagnostic using the 2021 pilot cities and cities never treated during the sample period. The 2020 wave is the reference period. We interact the 2021-cohort indicator with the 2014, 2016, 2018, and 2022 survey-year indicators while retaining the same individual and city controls, individual and year fixed effects, and city-clustered standard errors as in the baseline model. The diagnostic sample contains 841 observations, 318 individuals, and 65 cities.

**Figure 1:**
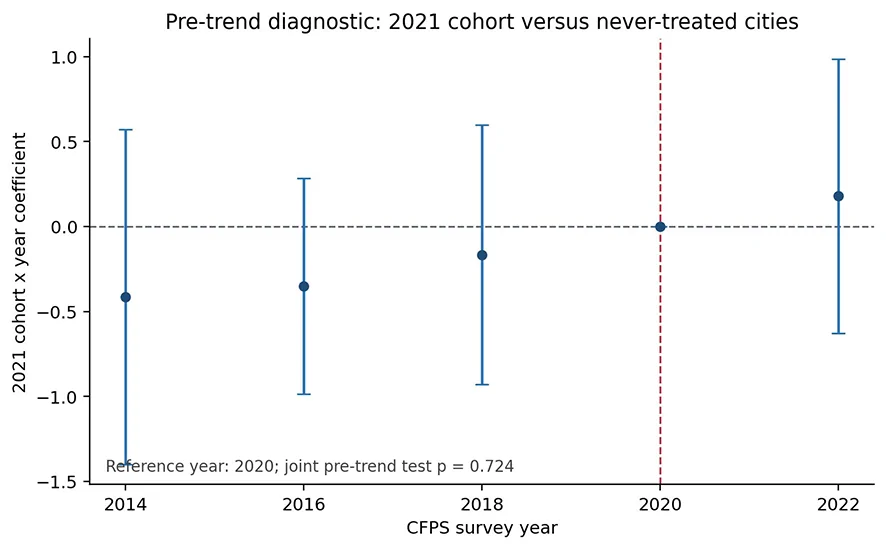
Pre-trend diagnostic for the 2021 pilot cohort relative to never-treated cities; 2020 is the reference year and bars show 95% confidence intervals.

Relative to 2020, the pre-policy coefficients are −0.4136 (SE = 0.4934; 95% CI: −1.3994–0.5722) for 2014, −0.3512 (SE = 0.3171; 95% CI: −0.9847–0.2824) for 2016, and −0.1664 (SE = 0.3822; 95% CI: −0.9299–0.5972) for 2018. A joint test does not reject that the three coefficients equal zero (F(3, 64) = 0.4419; *p* = 0.7239). Figure 1 therefore provides evidence consistent with comparable pre-reform trends for this diagnostic sample, although failure to reject the joint null should not be interpreted as proof that the parallel-trends assumption necessarily holds.

### 4.3 Robustness checks

#### 4.3.1 PSM-DID

DRG/DIP pilot cities were not selected completely at random and may differ systematically from non-pilot cities in economic development, urbanization, financial services, and informatization. We therefore estimate city-level propensity scores from pre-policy GDP per capita, urbanization, financial development, and informatization and combine nearest-neighbor matching with DID. The preferred specification uses 1:2 nearest-neighbor matching without replacement and a caliper of 0.3 standard deviations of the logit propensity score. In the unmatched 2018 city cross-section, the mean and maximum absolute standardized mean differences for these four covariates are 0.3082 and 0.4178. After matching in the preferred PSM024 specification, they decline to 0.0481 and 0.0866. We further vary common-support restrictions, winsorization, the number of neighbors, caliper widths, and matching covariates, producing eight specifications in which every post-match absolute standardized mean difference is below 0.10.

Across the matching and sample-treatment specifications in Table 3, the DID coefficient ranges from 0.4799 to 0.6074 and is consistently significant at the 5% level. In the preferred PSM024 specification, the coefficient is 0.5511 with a city-clustered standard error of 0.2311. The mean absolute standardized difference ranges from 0.0426 to 0.0688, and the maximum is below 0.10 in every specification. Balance between treated and control cities therefore improves substantially, while the conclusion that DRG/DIP reform increases household financial product allocation remains unchanged.

**Table 3:** PSM-DID robustness checks: strictly balanced specifications.

Panel A				
**Variables**	**PSM024**	**PSM084**	**PSM054**	**PSM053**
DRG/DIP reform (DID)	0.5511^**^	0.5511^**^	0.5700^**^	0.4799^**^
	(0.2311)	(0.2311)	(0.2217)	(0.2244)
Matching covariates	city4	city4	city4	city4
Sample treatment	No winsorization	5%−95% winsorization	Common support	Common support
Matched neighbors	2	2	2	2
Caliper	0.3	0.3	0.3	0.2
Without replacement	YES	YES	YES	YES
Individual/city controls	YES	YES	YES	YES
Individual/year fixed effects	YES	YES	YES	YES
City-clustered standard errors	YES	YES	YES	YES
Observations	804	804	827	841
Cities	51	51	51	48
Mean |SMD|	0.0481	0.0481	0.0517	0.0567
Max |SMD|	0.0866	0.0866	0.0951	0.0975
Within R-squared	0.0565	0.0565	0.0574	0.0505
Panel B				
**Variables**	**PSM023**	**PSM083**	**PSM059**	**PSM148**
DRG/DIP reform (DID)	0.5195^**^	0.5195^**^	0.5407^**^	0.6074^**^
	(0.2381)	(0.2381)	(0.2403)	(0.2776)
Matching covariates	city4	city4	city4	city6
Sample treatment	No winsorization	5%−95% winsorization	Common support	Common support
Matched neighbors	2	2	3	3
Caliper	0.2	0.2	0.3	0.2
Without replacement	YES	YES	YES	YES
Individual/city controls	YES	YES	YES	YES
Individual/year fixed effects	YES	YES	YES	YES
City-clustered standard errors	YES	YES	YES	YES
Observations	778	778	843	654
Cities	48	48	52	45
Mean |SMD|	0.0592	0.0592	0.0426	0.0688
Max |SMD|	0.0974	0.0974	0.0951	0.0928
Within R-squared	0.0605	0.0605	0.0577	0.0691

City-clustered robust standard errors are in parentheses. city4 includes pre-policy city GDP per capita, urbanization, financial development, and informatization; city6 additionally includes GDP growth and industrial structure. In the unmatched 2018 city cross-section for city4, mean |SMD| = 0.3082 and max |SMD| = 0.4178. In the preferred PSM024 specification after matching, mean |SMD| = 0.0481 and max |SMD| = 0.0866. For all eight matched specifications, the absolute standardized mean difference of every covariate is below 10%. ^**^ denotes statistical significance at the 5% level.

#### 4.3.2 Inverse hyperbolic sine transformation

The baseline dependent variable is ln(1+total financial product value). To test whether the estimates depend on this transformation, we replace it with the inverse hyperbolic sine of the original value. This transformation retains zero observations, compresses the right tail, and approximates the natural logarithm for large values.

Column (2) of Table 4 shows that the DID coefficient under the inverse hyperbolic sine transformation is 0.3978 and significant at the 1% level. Its sign, magnitude, and statistical significance are very close to the baseline estimate, indicating that the main conclusion is not driven by the logarithmic transformation.

**Table 4:** Robustness to the inverse hyperbolic sine transformation.

Variables	(1) ln(1+financial product value)	(2) asinh (financial product value)
DRG/DIP reform (DID)	0.3952^***^	0.3978^***^
	(0.1301)	(0.1306)
Individual controls	YES	YES
City controls	YES	YES
Individual fixed effects	YES	YES
Year fixed effects	YES	YES
City-clustered standard errors	YES	YES
Observations	1,689	1,689
Cities	79	79
Individuals	641	641
Within R-squared	0.0251	0.0253

City-clustered robust standard errors are in parentheses. ^***^ denotes statistical significance at the 1% level.

#### 4.3.3 City-level randomization placebo test

To assess whether the baseline result could be generated by unobserved city shocks or random policy assignment, we randomly reassign policy years among the 79 cities in the baseline sample while preserving the numbers of treated cities in the 2019 and 2021 cohorts. Repeating the estimation 5,000 times produces an empirical distribution of placebo DID coefficients. If the baseline result were largely random, the actual coefficient would not lie far from this distribution.

**Figure 2:**
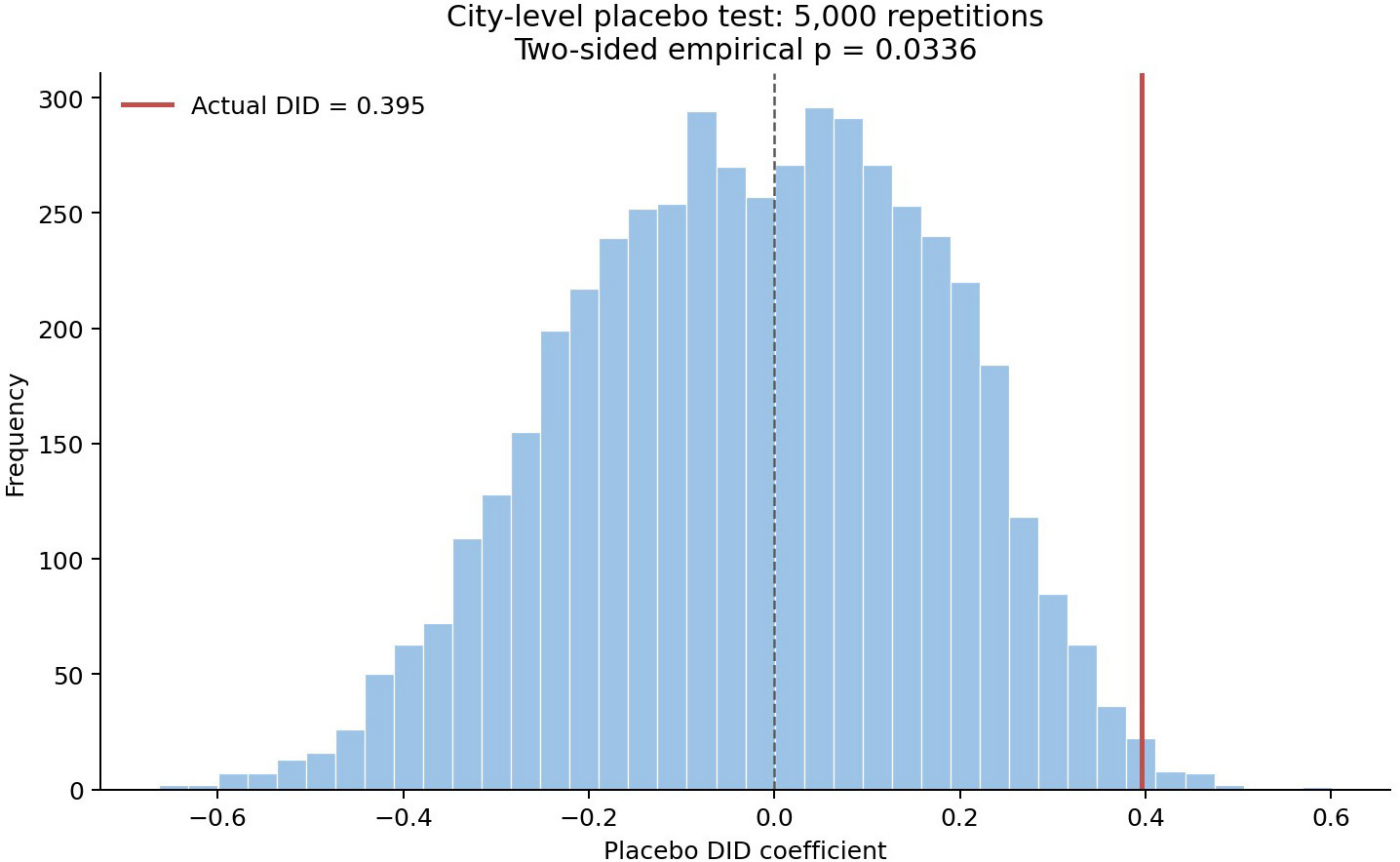
City-level randomization placebo test based on 5,000 permutations.

As shown in Table 5 and Figure 2, the 5,000 placebo estimates have a mean of −0.0229 and are concentrated around zero, whereas the actual DID coefficient of 0.3952 lies in the right tail. The two-sided empirical *p*-value is 0.0336, meaning that a random assignment produces a coefficient with an absolute value at least as large as the actual estimate in approximately 3.36% of repetitions. The baseline effect is therefore unlikely to be driven by random policy assignment or incidental city-level shocks.

**Table 5:** City-level randomization placebo test.

Statistic	City-level randomization result
Number of permutations	5,000
Actual DID coefficient	0.3952
Mean placebo coefficient	−0.0229
SD of placebo coefficients	0.1949
Two-sided empirical p-value	0.0336

The two-sided empirical *p*-value is the share of placebo coefficients whose absolute value is at least as large as the absolute value of the actual DID coefficient.

### 4.4 Heterogeneity analysis

The baseline model identifies an average reform effect, but households may differ in their ability to translate institutional change into financial decisions. To avoid defining groups with post-treatment values, we use each household's most recent observed pre-policy status. Spouse status and internet use are binary indicators, and total household expenditure is divided at the sample median of pre-policy ln(1+expenditure). We interact DID with each grouping indicator and test the between-group difference directly.

Panel A of Table 6 shows a DID effect of 1.1359 for households without a spouse, significant at the 1% level, and an effect of 0.3170 for households with a spouse, significant at the 5% level. The difference is −0.8189 and significant at the 1% level, indicating a stronger response among households without a spouse. Such households may face fewer shared-decision constraints and household responsibilities and can therefore adjust their portfolios more flexibly when expectations of medical spending and the institutional environment change.

**Table 6:** Heterogeneity analysis.

Panel A: spouse status		
**Variables**	**No spouse**	**Has spouse**
DRG/DIP reform (DID)	1.1359^***^	0.3170^**^
	(0.2095)	(0.1303)
Individual/city controls	YES	YES
Individual/year fixed effects	YES	YES
City-clustered standard errors	YES	YES
Observations	1,539	1,539
Cities	74	74
Within R-squared	0.0349	0.0349
Panel B: total household expenditure		
**Variables**	**Lower-expenditure households**	**Higher-expenditure households**
DRG/DIP reform (DID)	0.6893^***^	0.3011^**^
	(0.1750)	(0.1318)
Individual/city controls	YES	YES
Individual/year fixed effects	YES	YES
City-clustered standard errors	YES	YES
Observations	1,539	1,539
Cities	74	74
Within R-squared	0.0296	0.0296
Panel C: internet use		
**Variables**	**No internet use**	**Internet use**
DRG/DIP reform (DID)	−0.1033	0.4231^***^
	(0.2519)	(0.1468)
Individual/city controls	YES	YES
Individual/year fixed effects	YES	YES
City-clustered standard errors	YES	YES
Observations	1,420	1,420
Cities	72	72
Within R-squared	0.0279	0.0279

City-clustered robust standard errors are in parentheses. ^***^ and ^**^ denote statistical significance at the 1% and 5% levels, respectively. Groups are defined by the most recent pre-policy status. Total expenditure is split at the sample median of pre-policy ln(1+household expenditure). Between-group differences reported in the text are estimated from the same interaction models.

Panel B reports a coefficient of 0.6893 for lower-expenditure households and 0.3011 for higher-expenditure households, significant at the 1% and 5% levels, respectively. The difference between the high- and low-expenditure groups is −0.3882 and significant at the 1% level. Reform therefore has a larger marginal effect among lower-expenditure households, which may be more sensitive to medical cost uncertainty and payment rules. When reform improves expected medical expenditure, the resources and willingness available for formal financial products rise more sharply in this group.

Panel C shows that the coefficient for households without pre-policy internet use is −0.1033 and statistically insignificant, whereas the coefficient for internet users is 0.4231 and significant at the 1% level. The difference of 0.5264 is significant at the 5% level. Digital access thus appears to be an important condition for obtaining policy information, understanding financial products, and translating institutional expectations into portfolio decisions.

### 4.5 Mechanism analysis

We use Jiang's two-step approach as an exploratory assessment of possible channels. The first step estimates the total reform effect on household financial product value. The second step separately uses household consumption expenditure, government intervention, and industrial structure upgrading as dependent variables to determine whether reform moves each theoretically proposed channel. The mechanism variable is not reintroduced into the outcome equation, and no conventional mediation share is calculated. These regressions therefore provide channel-consistent evidence, not identification of a causal mediated effect.

Column (2) of Table 7 reports a coefficient of 0.1786 for household consumption expenditure, significant at the 5% level. The increase is consistent with the proposition that more predictable medical payment rules may improve household expectations. The simultaneous rise in consumption and financial product holdings is also consistent with households relying less exclusively on liquid precautionary reserves, but the two-step design does not establish that consumption causally mediates the portfolio effect.

**Table 7:** Mechanism tests using Jiang's two-step approach.

Variables	Financial product value	Consumption expenditure	Government intervention	Industrial upgrading
DRG/DIP reform (DID)	0.3952^***^	0.1786^**^	−0.3730^***^	0.1593^***^
	(0.1301)	(0.0855)	(0.1257)	(0.0591)
Individual controls	YES	YES	YES	YES
City controls	YES	YES	YES	YES
Individual fixed effects	YES	YES	YES	YES
Year fixed effects	YES	YES	YES	YES
City-clustered standard errors	YES	YES	YES	YES
Observations	1,689	1,578	1,689	1,231
Cities	79	79	79	73
Individuals	641	601	641	494
Within R-squared	0.0251	0.0715	0.4590	0.4269

Household consumption expenditure, government intervention, and industrial structure upgrading are logged and standardized. City-clustered robust standard errors are in parentheses. ^***^ and ^**^ denote statistical significance at the 1% and 5% levels, respectively. No Sobel test or Benjamini–Hochberg adjustment is applied. The two-step estimates are interpreted as exploratory channel-consistent evidence rather than causal mediation effects.

Column (3) reports a coefficient of −0.3730 for government intervention, significant at the 1% level. Because the indicator measures the degree of intervention, its decline should not be interpreted as government withdrawal from medical protection. It is consistent with a transition from item review and direct administrative control toward constraints based on disease groups, disease categories, and global budget rules. The result supports the proposed institutional-certainty interpretation but does not by itself identify a causal mediation path.

Column (4) reports a coefficient of 0.1593 for industrial structure upgrading, significant at the 1% level. The estimate is consistent with the possibility that payment reform accompanies healthcare informatization, health services, and related modern services. Taken together, the three mechanism outcomes move in directions predicted by the theoretical framework. We interpret them as exploratory channel-consistent evidence rather than as additive or causal mediation effects.

## 5 Discussion

### 5.1 Main findings

Medical insurance payment is a core institution of the healthcare system. It affects provider incentives and insurance-fund sustainability and may also shape household behavior through expectations of medical expenditure. Using five CFPS waves and a city-level quasi-natural experiment, we find that DRG/DIP reform is associated with a higher total value of household financial products. The estimate remains positive under individual and year fixed effects, multidimensional controls, PSM-DID, an inverse hyperbolic sine outcome, exclusion of cross-city movers, and 5,000 city-level placebo permutations. A cohort-specific diagnostic also finds no joint evidence of differential pre-reform trends for the 2021 cohort relative to never-treated cities. The consumption, government-intervention, and industrial-upgrading regressions are consistent with the proposed theoretical channels, but they do not establish causal mediation. Heterogeneity by spouse status, household expenditure, and internet access indicates that the capacity to translate institutional change into financial decisions is uneven. From a public-health perspective, these findings suggest that healthcare financing rules may influence household welfare and financial resilience in addition to provider behavior and service efficiency.

### 5.2 Policy implications

First, medical insurance payment reform should be deepened steadily to realize its cross-domain positive externalities. DRG/DIP reform is not only a tool for containing rapid medical cost growth and improving service efficiency; by stabilizing expectations and reducing precautionary saving motives, it can also promote household financial asset allocation. Policymakers should continue expanding and deepening the reform, refine disease-group and disease-category classifications and payment standards, and improve the standardization and transparency of settlement. Regions already using actual payment should strengthen dynamic monitoring and evaluation and adjust weights and thresholds in a timely manner to prevent unintended consequences such as lower quality or avoidance of severe cases. Regions still in transition should strengthen information systems and data accumulation to support scientifically determined payment standards. Policy evaluation should also incorporate effects on household consumption, saving, and financial welfare so that the broader social value of payment reform is recognized.

Second, medical insurance governance should continue shifting from administrative intervention toward standardized, rule-based management to strengthen predictability. The mechanism results suggest that the decline in government intervention represents a transition from item review, direct cost control, and ex post supervision toward classification, weight calculation, and global budget constraints. Policymakers should reduce temporary policy changes and discretionary space and establish regular revision and advance disclosure of classification schemes, payment standards, and settlement rules. Budget management and information disclosure should also be strengthened, with regular publication of disease-level cost composition, payment shares, and patient out-of-pocket burdens. Better information would allow households to assess medical expenditure risk more accurately, reduce excessive precautionary reserves, and allocate funds more efficiently between consumption and formal financial products.

Third, implementation should be adapted to local conditions and to households' unequal capacity to absorb policy benefits. Households without a spouse, lower-expenditure households, and internet users are better able to translate institutional change into financial decisions. The weaker response among households with a spouse and higher-expenditure households may reflect shared-decision constraints and rigid medical budgets. Supplementary major-illness insurance, links to commercial health insurance, and medical assistance could further share high-cost risk and release room for financial allocation. The insignificant effect among households without internet access highlights the digital divide. Community-level policy communication, offline financial education, and face-to-face guidance through primary medical institutions and community service centers can reduce informational disparities. Within a unified national framework, local governments should also be allowed to adjust classification detail and implementation pace according to medical resources and household economic conditions, avoiding a one-size-fits-all approach.

Fourth, coordination among medical payment reform, industrial policy, financial policy, and informatization policy should be strengthened. The industrial structure mechanism shows that DRG/DIP reform operates beyond the healthcare system by encouraging healthcare informatization, health management, commercial insurance, medical big data, and related modern services, which can improve urban employment, income, and financial service supply. Cross-departmental coordination should integrate payment reform with regional medical center development, health-industry planning, and inclusive finance, guiding capital, technology, and labor toward more efficient health and modern service sectors. Subject to data security and privacy protection, the disease-cost and service information generated by reform can be connected orderly with financial infrastructure. Financial institutions could then develop prudent products aligned with households' expected medical expenditure, providing suitable investment channels after precautionary medical reserves decline.

### 5.3 Limitations

Several limitations warrant caution. First, pilot designation is not random and may remain correlated with unobserved time-varying city characteristics; fixed effects, matching, and placebo tests reduce but do not eliminate this concern. Second, policy exposure is assigned from current-wave prefecture codes. Cross-city migration is rare in the baseline sample and excluding movers produces a similar estimate, but location measurement error cannot be ruled out completely. Third, FT201 is a self-reported aggregate value and does not permit a consistent decomposition of portfolio composition across all five waves. Fourth, the two-step mechanism regressions establish whether proposed channel variables respond to reform, not whether they causally mediate the portfolio effect. Finally, the findings are most directly applicable to the observed CFPS households and pilot cities and should be generalized to other populations or payment settings with caution.

## Data Availability

The CFPS data analyzed in this study are available from the Institute of Social Science Survey, Peking University, subject to its data-use conditions. The city-level policy and economic indicators were compiled from official and statistical sources. Further inquiries can be directed to the corresponding author.
